# Epoxy Phase-Change Materials Based on Paraffin Wax Stabilized by Asphaltenes

**DOI:** 10.3390/polym15153243

**Published:** 2023-07-29

**Authors:** Svetlana O. Ilyina, Anna V. Vlasova, Irina Y. Gorbunova, Nikolai I. Lukashov, Michael L. Kerber, Sergey O. Ilyin

**Affiliations:** 1 A.V. Topchiev Institute of Petrochemical Synthesis, Russian Academy of Sciences, 29 Leninsky Prospect, 119991 Moscow, Russia; 2 Department of Plastics Processing Technology, D. Mendeleev University of Chemical Technology of Russia, 9 Miusskaya Square, 125047 Moscow, Russia

**Keywords:** phase-change materials, epoxy resin, paraffin wax, asphaltenes, Pickering emulsions, rheology

## Abstract

The usual problem of meltable phase-change agents is the instability in their form upon heating, which can be solved by placing them into a continuous polymer matrix. Epoxy resin is a suitable medium for dispersing molten agents, but it is necessary to make the obtained droplets stable during the curing of the formed phase-change material. This work shows that molten paraffin wax forms a Pickering emulsion in an epoxy medium and in the presence of asphaltenes extracted from heavy crude oil. Theoretical calculations revealed the complex equilibrium in the epoxy/wax/asphaltene triple system due to their low mutual solubility. Rheological studies showed the viscoplastic behavior of the obtained dispersions at 25 °C, which disappears upon the heating and melting of the paraffin phase. Wax and asphaltenes increased the viscosity of the epoxy medium during its curing but did not inhibit cross-linking or reduce the glass transition temperature of the cured polymer. As a result of curing, it is possible to obtain phase-change materials containing up to 45% paraffin wax that forms a dispersed phase with a size of 0.2–6.5 μm. The small size of dispersed wax can decrease its degree of crystallinity to 13–29% of its original value, reducing the efficiency of the phase-change material.

## 1. Introduction

Ensuring the welfare of a growing population requires an increase in the production of economic goods and hence an increase in resource consumption. Trends in the global economy are such that energy consumption is growing [1,2]. At the same time, reservoirs of fossil fuels are limited, while the demand for petroleum production and consumption is constantly increasing [3]. Moreover, the increased consumption of fossil fuels is leading to higher concentrations of carbon dioxide, global warming, and a higher carbon footprint. This determines the relevance of the development of energy-saving technologies both in industrial production and in the residential sector.

One of the modern innovative ways to increase energy efficiency is via phase-change materials (PCMs) [4]. PCMs can have applications in many areas of activities in life [5,6,7,8,9]. The most widespread application of PCMs is through maintaining a narrow temperature range close to the melting and crystallization temperatures of the phase-change agent. This allows the rational use of heat and saves energy required for building heating [10,11]. In addition, it is possible to use PCMs in specific fields, e.g., as a “smart” coolant for chemical reactors or mechanical energy storage systems [12,13]. A wide variety of different phase-change agents is suitable for creating PCMs, which allows for selecting the right combination of components with the required operating temperature range for their use in specific thermal conditions [14,15].

It is common to distinguish organic and inorganic phase-change materials and their eutectic combinations [16,17,18,19]. The main feature of phase-change materials is the use of their latent heat energy, where heat is released or absorbed during the transition between the liquid and solid aggregate states. This heat energy is much higher than that emitted or absorbed due to a change in the material’s temperature [20,21,22]. However, the problem with PCMs is the loss of their shape due to the transition of the phase-change agent to a molten low-viscous state, which is not permissible in all applications [23,24,25]. The solution to this problem is to cover the phase-change agent with a shell [26,27,28,29,30,31,32] or place it into a continuous solid matrix [33,34,35,36,37].

Paraffin wax is the most common crystallizing phase-change agent for creating PCMs [38,39,40,41,42]. It is an easily available and low-cost substance that is chemically stable and nontoxic with a high heat storage capacity in a relatively narrow temperature range. Polymers and organic gels are used mainly as matrices for creating PCMs [43,44,45,46] as they can act as a supporting structure that allows for keeping their shape. In the case of paraffin wax, thermoplastic polymer matrices have previously been used to impart form stability, e.g., high-density polyethylene (HDPE) to melt and mix with wax at high temperatures [47]. In the temperature range below the crystallization point of HDPE and above the melting point of the wax, wax is liquid, while HDPE is solid. This allows for creating a stable solid-like PCM that holds its shape without the leakage of liquid wax due to the solid state of HDPE. Another shape-stable PCM can be formed by mixing wax with a thermoplastic styrene–butadiene–styrene elastomer, in which its thermal conductivity can be increased significantly with the addition of graphite [48].

However, the method of combining phase-change substances with thermoplastics has drawbacks. The main problem in their mixing is the high viscosity of polymer melts compared to paraffin wax, which requires a highly intense stirring that is unachievable in practice due to the high-molecular-weight nature of polymers and the emergence of its rubbery-like behavior [49]. It is advisable to use thermosetting polymers, which have a much lower viscosity and enable a uniform distribution of the phase-change agent without difficulties in the preparation of the emulsion. The most readily available thermosetting plastics suitable for this purpose are epoxy resins, which have a low viscosity and are cured at a temperature of interest by choosing a hardener [50,51,52,53].

The maximum possible concentration and the eventual distribution of a phase-change agent are necessary to achieve the highest energy efficiency of the finished PCM. In the case of paraffin wax, it is essential to avoid the formation of its aggregates and large particles that are prone to sedimentation caused by density differences. For sedimentation resistance, it is necessary to disperse wax carefully to obtain little molten droplets or fine crystalline particles of wax. However, big crystalline wax particles are almost impossible to break up in a liquid epoxy medium by stirring, while small particles of wax tend to aggregate strongly. On this basis, it would be easiest to disperse the material when the epoxy/wax blend is heated. The wax would melt, and the viscosity of the epoxy resin would decrease, which would facilitate the dispersion process greatly. In addition, the dispersion of wax in the liquid state would potentially allow for a higher content of wax in the epoxy medium because the maximum volume fraction of droplets in the emulsion would be higher than that of solid particles in the suspension, reaching more than 90% [54,55]. An extra advantage of highly concentrated emulsion-type pre-PCMs compared to suspended ones is their ability to flow, which allows for creating products with intricate designs using casting frames.

However, paraffin wax is non-polar and hence low-compatible with more polar epoxy resin. Consequently, melted wax droplets in an epoxy medium coalesce quickly with each other, causing the layering of the emulsion. Therefore, there is a need to stabilize the wax droplets. The problem is that most surfactants serve to stabilize emulsions with a non-polar organic or polar protic (water) medium. Epoxy resins are relatively polar compounds with polar and non-polar functional groups but do not form intermolecular hydrogen bonds. It is problematic to select an effective surfactant suitable for dissolution in the epoxy medium and subsequent adsorption on the surface of molten wax droplets to prevent their coalescence. The alternative method for stabilizing emulsion is using micro- or nanoparticles to form a Pickering emulsion [56]. In Pickering emulsions, solid particles adsorb on the surface of the droplets, thus creating a steric barrier, which leads to a more effective stabilization of the droplets against coalescence than with surfactants [57]. Moreover, solid particles can form a percolation structure even at low concentrations, giving emulsions a resistance to sedimentation and superstability [58,59].

Silica, montmorillonite, graphene oxide, chitosan, cyclodextrin, hydroxyapatite, and other nanoparticles and nanotubes are used as Pickering emulsifiers [60,61]. Moreover, solid particles can improve the thermal conductivity of PCMs as well as stabilize their phase-change agents [62]. For example, graphite particles create a Pickering emulsion based on epoxy resin and paraffin wax for PCM with higher thermal conductivity [57]. At the same time, other carbon-containing particles with potentially high thermal conductivity have attracted the attention of researchers in recent years as stabilizers of Pickering emulsions: asphaltenes [63,64]. Asphaltenes are a heavy fraction of crude oil, remaining after oil deasphalting or refining [65]. They require rational disposal, and there are ways to use asphaltenes for producing plastics [66,67,68,69], bitumens [70,71], and carbon fibers [72]. Asphaltenes are a broad spectrum of chemicals having hydrophobic hydrocarbon structures with some hydrophilic functional groups [73,74]. Asphaltenes are surface-active substances because they can adsorb on the water–oil interface, stabilizing emulsions [75,76]. Over time, asphaltene molecules assemble into a monolayer about 2–9 nm thick, forming a firm elastic film that stabilizes the emulsion droplets [77]. The ability of asphaltenes to emulsify water–oil systems causes exploitation and manufacturing problems in crude oil production [78]. In this regard, a method for stabilizing epoxy emulsions with asphaltenes can be a reasonable opportunity to use their surface activity practically for obtaining a stable paraffin wax/epoxy resin emulsion, which is hard to produce with conventional surfactants.

This work aimed to obtain and study a phase-change material based on an emulsion of paraffin wax in epoxy resin using asphaltenes as a stabilizer. Firstly, we will calculate theoretically binary phase diagrams for pairs of these compounds using the Flory–Huggins theory to evaluate the phase state of epoxy blends and the mutual solubility of their components at different temperatures. Then, we will measure the flow curves and viscoelasticity of the uncured blends to estimate the effect of paraffin wax on their viscosity and structure. Afterward, we will assess the effect of wax addition on the cross-linking of the blends by measuring the viscosity and heat release during their non-isothermal curing. Finally, we will investigate the cured blends via dynamic mechanical analysis and differential scanning calorimetry to examine the effect of paraffin wax on their elastic modulus, glass transition temperature, and effectiveness as phase-change materials, including evaluating blends’ morphologies via electron microscopy.

## 2. Materials and Methods

### 2.1. Materials

Diglycidyl ether of bisphenol A (D.E.R.™ 330, Dow Chemical, Midland, MI, USA), having an epoxide equivalent weight of 180.5 g/eq and an epoxide percentage of 23.8%, was used as an epoxy resin. The hardener was 4,4′-diaminodiphenyl sulfone (Sigma-Aldrich, Steinheim, Germany) with an amine hydrogen equivalent weight of 62.1 g/eq. The resin/hardener ratio was stoichiometric: 74.4/25.6 wt%/wt%.

Paraffin wax (P-2, JSC “Yaroslavl paraffin-waxes plant”, Rostov, Russia) with a number-average molecular weight (*M*_n_) of 563 g/mol, a dispersity (*M*_w_/*M*_n_) of 1.05, and a melting point of 54.4 °C was a phase-change agent. Its mass fractions in mixtures of resin, hardener, and asphaltenes were 0, 15, 25, 35, and 45 wt%.

Asphaltenes were obtained by adding a 15-fold excess of hexamethyldisiloxane (ECOS-1, Moscow, Russia) to heavy crude oil from the Ashalcha oilfield [79,80] (Almetyevsk, Russia), as was described earlier [81]. Their weight-average molecular weight (*M*_w_), dispersity, and softening point were 828 g/mol, 1.14, and 71 °C, respectively. This asphaltene blend consisted of 49.7% resins and 19.3% heptane-insoluble asphaltenes, while the remaining part was an admixture of heavy aromatic compounds and crystallizing saturates [82]. In all mixtures, the asphaltene mass fraction was ten times less than that of paraffin wax. Table 1 shows the final compositions of the prepared mixtures.

Mixing of all components was performed using an Ultra-Turrax T18 disperser (IKA, Staufen, Germany) for 1 min after preheating to 100 °C. Then, part of the mixed blends was cooled to 25 °C for rheological and calorimetric tests, while the remaining amount was placed in a thermal oven at 180 °C for curing for 3 h.

### 2.2. Methods

Rheological properties were investigated on a Discovery HR-2 rotational rheometer (TA Instruments, New Castle, DE, USA). Flow curves and frequency dependences of storage (*G*′) and loss (*G*″) moduli of uncured samples were obtained at 25 °C using a plate–plate measuring unit with a plate diameter of 8 mm and interplate gap of 0.5 mm. The flow curves of the same samples at 100 °C were measured on the cone–plate unit with a plate diameter of 40 mm and an angle between the conical surface and the plate of 2°. Viscosity (*η*) was estimated in a stepwise mode of increasing the shear rate ($\dot{\gamma }$) from 10^−3^ s^−1^ to 1000 s^−1^ with a measurement time for each shear rate value of 30 s. Viscoelastic properties were studied at a small strain amplitude of 0.1% in the angular frequency range of 0.0628–628 rad/s. For analyzing the temperature dependence of the effective viscosity in the temperature range of 25–220 °C, a plate–plate measuring unit was used with plates’ diameters of 8 mm. In this case, measurements were carried out at a constant shear rate of 1 s^−1^ and a heating rate of 2 °C/min. We performed rheological tests two or three times to ensure the reproducibility of the obtained curves. The equations for calculating the rheological characteristics can be found elsewhere [83].

The viscoelastic properties of the cured samples were investigated via dynamic mechanical analysis (DMA) on an Eplexor 9 analyzer (Netzsch, Selb, Germany) using the three-point bending method. The specimens were bars having a width of 12.4 mm, a thickness of 2.8 mm, and a length of 40 mm (the distance between the two outermost clamps was 30 mm). The tests were at a deformation frequency of 1 Hz and a contact force amplitude of 0.8 N in the temperature range of 30–250 °C at a heating rate of 2 °C/min.

Differential scanning calorimetry (DSC) was performed on an MDSC 2920 calorimeter (TA Instruments) in an argon medium. The curing of compositions was at a heating rate of 2 °C/min in the temperature range from 25 °C to 280 °C. The cured samples were tested by increasing or decreasing the temperature at a rate of 10 °C/min in the range of 10–240 °C. The accuracy of measuring transitions’ temperatures was ±0.2 °C, while the maximum relative error in determining their enthalpies did not exceed 5%. Although multiple heating/cooling cycles may degrade the performance of phase-change materials [84,85,86,87,88,89], we tested the samples by repeating the heating/cooling cycle three times only to evaluate their potential qualitatively without multiple long-term tests. We used these tests to calculate the average temperatures and enthalpies of transitions.

Scanning electron microscopy (SEM) of cured samples was conducted on a Phenom XL G2 microscope (Thermo Fisher Scientific, Eindhoven, the Netherlands) at an accelerating voltage of 15 kV and a pressure of 60 Pa. Preliminarily, a 5 nm silver layer was applied to the surface of the samples by ion-plasma sputtering on the 108 Auto device (Cressington Scientific Instruments, Watford, UK). Calculation of droplet size distribution was performed in ImageJ software (National Institutes of Health, Bethesda, MD, USA).

## 3. Results and Discussion

### 3.1. Phase State of Asphaltenes/Paraffin Wax/Epoxy Resin Blends

The mission of asphaltenes is to act as surfactants that stabilize molten wax droplets during blend preparation and high-temperature curing. On this basis, asphaltenes should be at least partially soluble in the epoxy medium. In addition, although asphaltenes are insoluble in low-molecular-weight aliphatic hydrocarbons (e.g., heptane), they can dissolve in paraffin wax because of its better affinity to asphaltenes (closer solubility parameters: 15.3 MPa^0.5^ for heptane [90], 16.7 MPa^0.5^ for paraffin wax [91], and 19.6 MPa^0.5^ for asphaltenes [92,93]). Moreover, solubility can become higher with increasing temperature, which can lead to the partial solubility of epoxy resin and paraffin wax, which are insoluble in each other at room temperature. Thus, a complex equilibrium between coexisting phases can be in a multi-component epoxy system, being not appropriate to study using conventional methods such as the cloudy-point method. At the same time, knowledge of the mutual solubility of the components helps evaluate what happens in the system during mixing and heating for subsequent curing.

The mutual solubility of two substances can be estimated based on the equality of the free energy increments for each substance in coexisting phases [94,95,96,97]:

(1) $$\Delta \bar{G_{i}^{\prime }}=\Delta \bar{G_{i}^{\prime \prime }},$$

where $\Delta \bar{G_{i}^{\prime }}$ and $\Delta \bar{G_{i}^{\prime \prime }}$ are the increments of the partial molar Gibbs free energy of the *i*-th component in the first and second phases, respectively. For a two-component mixture, the increment of partial molar free energy can be calculated using the following equation:

(2) $$\Delta \bar{G_{1}^{\prime }}=RT\left(ln\varphi_{1}+\frac{V_{m,2}-V_{m,1}}{V_{m,2}}\varphi_{2}+\chi_{12}\varphi_{2}^{2}\right),$$

where *φ*_1_ and *φ*_2_ are the volume fractions of the 1st and 2nd components, respectively, while *V*_m,1_ and *V*_m,2_ are their molar volumes, *R* is the universal gas constant, *T* is the thermodynamic temperature, and *χ*_12_ is the Flory–Huggins interaction parameter. For the second component ($\Delta \bar{G_{2}^{\prime }}$), the form of the equation does not change except for the corresponding indices.

The Flory–Huggins interaction parameter can be found from the Hildebrand solubility parameters of the mixture ingredients as follows:

(3) $$\chi_{12}=\frac{V_{m,1}{\left(\delta_{1}-\delta_{2}\right)}^{2}}{RT},$$

where *δ*_1_ and *δ*_2_ are the Hildebrand solubility parameters for the 1st and 2nd components. For the epoxy resin, the solubility parameter is 21.56 MPa^0.5^ [98,99], while the molar volume is 290.6 cm^3^·mol^−1^. Since paraffin wax has a similar chemical structure to polyethylene, we decided to use the solubility parameter of polyethylene in calculations (16.7 MPa^0.5^ [100]), while the wax’s molar volume is equal to 625.56 cm^3^·mol^−1^ based on its molecular weight and density (approximately 0.9 g/cm^3^). In turn, the solubility parameter of asphaltenes is about 19.6 MPa^0.5^ [92,93], and the molar volume is 704 cm^3^·mol^−1^.

**Figure 1:**
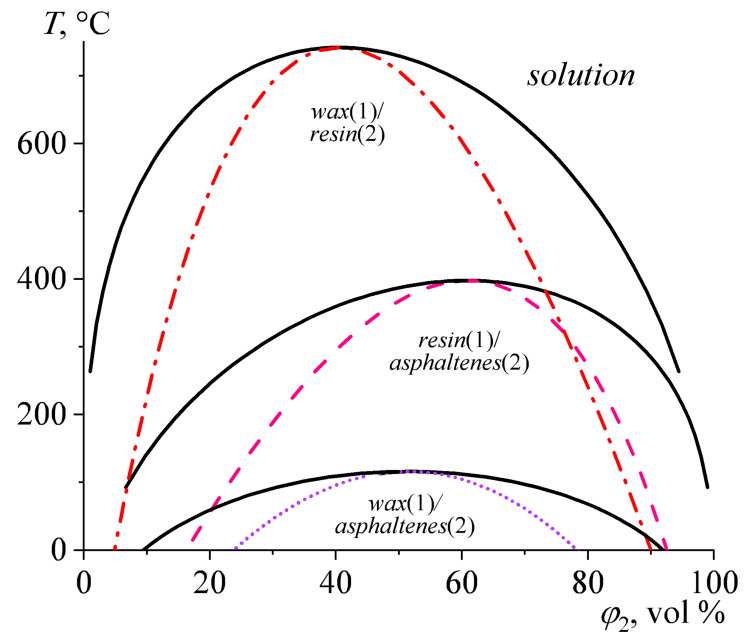
Calculated phase diagrams for blends of epoxy resin with paraffin wax, epoxy resin with asphaltenes, and paraffin wax with asphaltenes. The solid and dashed lines indicate the binodal and spinodal curves, respectively. In all cases, homogeneous solutions locate at higher temperatures relative to these curves, while two-phase systems are lower.

The above data have allowed for calculating the binodal lines in the first approximation using Equations (1)–(3) (marked in Figure 1 with solid black lines). The calculations assumed that *V*_m_ and *δ* change equally with temperature for all components. In addition, it is also possible to calculate spinodal lines, where the second derivative of the Gibbs free energy with respect to the components’ volume fractions takes a zero value [101], as follows:

(4) $$\frac{d^{2}\Delta G}{d\varphi_{1}d\varphi_{2}}=0.$$

In the case of a two-component solution, the Flory–Huggins equation for free energy is as follows [102,103]:

(5) $$\Delta G=RT\left(\frac{\varphi_{1}}{V_{m,1}}ln\varphi_{1}+\frac{\varphi_{2}}{V_{m,2}}ln\varphi_{2}+\chi_{12}\varphi_{1}\varphi_{2}\right).$$

In the combination, Equations (3)–(5) allow for obtaining the following expression for the spinodal lines:

(6) $$\frac{1}{V_{m,1}\varphi_{1}}+\frac{1}{V_{m,2}\varphi_{2}}-2\left(\frac{{\left(\delta_{2}-\delta_{1}\right)}^{2}}{RT}\right)=0,$$

the application of which made it possible to construct the spinodal lines marked by the dashed color lines in Figure 1.

The calculated upper critical solution temperature (UCST) for the epoxy resin/paraffin wax blend is 742 °C. Thus, the dissolution of 1 vol% of epoxy resin in molten paraffin wax requires a temperature of about 263 °C. The solubility of wax in epoxy resin at the same temperature is higher: about 5.5 vol%. Therefore, epoxy resin and wax are very low soluble in each other, even at very high temperatures. It means that the system will be heterogeneous at the curing temperature (180 °C), and the solubility of epoxy resin and wax can be assumed to be negligible.

The miscibility of epoxy resin and asphaltenes is slightly better. At a curing temperature of 180 °C, the maximum solubility of asphaltenes in epoxy resin is about 13 vol%. The solubility of epoxy resin in asphaltenes is worse under the same conditions: only 3.5 vol%. At a mixing temperature of 100 °C, the maximum solubility of asphaltenes in the resin is 6.5 vol%, whereas the resin in asphaltenes is as low as 1 vol%. The mass fraction of asphaltene in our mixtures is 1.5–4.5 wt% (see Table 1), corresponding to the asphaltenes’ mass fraction of 2.4–10.7 wt% relative to pure epoxy resin. The density of epoxy resin (1.16 g/cm^3^) is comparable to that of asphaltenes (about 1.1–1.2 g/cm^3^), making the volume and mass fractions interchangeable. Therefore, a part of the asphaltenes in the samples with high wax content is in an insoluble (dispersed) state when the system is stirred at 100 °C. However, all added asphaltenes should theoretically dissolve fully in the epoxy medium at a curing temperature of 180 °C.

The paraffin wax/asphaltenes system is of particular interest; asphaltenes, being amorphous compounds, can dissolve in wax, inhibit its crystallization, decrease the degree of crystallinity, and, thereby, reduce the efficiency of phase-change material. The paraffin wax/asphaltenes pair shows the highest mutual solubility compared to other binary combinations. Its UCST is 115.5 °C, meaning that asphaltenes and paraffin wax are fully miscible when the epoxy blend is curing. Under the curing temperature, the used amounts of asphaltenes also theoretically dissolve fully in the epoxy resin that is practically immiscible with the molten wax. It means that asphaltenes dissolve completely and distribute in some way between the two coexisting phases of the epoxy resin and molten wax. As the epoxy resin cures and its molecular weight grows, the asphaltenes will lose solubility in the epoxy medium, passing partially into the paraffin phase and additionally forming a new asphaltene phase in the epoxy medium. At 25 °C, the solubility of asphaltenes in paraffin wax is about 12 vol%, and the wax solubility in asphaltenes reaches 10.5 vol%. In turn, the mass fraction of asphaltenes in the epoxy blends is 10% of that of the paraffin wax (see Table 1). It means that when the cured epoxy blends cool down, all asphaltenes absorbed by the paraffin phase will remain dissolved. However, asphaltenes, even when dissolved in a continuous medium, are nanoparticles with sizes of about 1–2 nm [73]. Moreover, asphaltenes are usually nanoaggregated even in dilute solutions to particles of 2–3 nm, which, nevertheless, are soluble (or dispersed, based on different interpretations).

Thus, the selected asphaltene content (up to 4.5 wt%, Table 1) should completely dissolve into the curing blend at high temperatures, but the blend will stay biphasic. This is a positive factor because asphaltene molecules have nanoscale sizes and can act as a Pickering emulsifier [64]. Note, however, that questions like “whether dissolved molecules or aggregated molecules can be assumed to be particles according to their relatively large size” and “whether emulsions stabilized by dissolved large dense molecules or their aggregates having nanosize can be assumed to be Pickering emulsions” are debatable. Due to dissolution, the total number of asphaltenes as dispersed elements will increase, which should help stabilize the paraffin droplets. Negative factors are the better solubility of asphaltenes in paraffin wax than in epoxy resin and their high solubility in wax even at 25 °C. As a result, the paraffin wax phase can be enriched with asphaltenes, which may inhibit the wax crystallization. At the same time, the calculations do not include the presence of a hardener. On the one hand, the hardener has a high melting point (176 °C) and a crystalline nature, thereby having negligible solubility in both asphaltenes and paraffin wax. On the other hand, it dissolves in the epoxy resin under curing conditions, thus changing its thermodynamic characteristics (solubility parameters) and either improving or worsening its miscibility with asphaltenes and paraffin wax.

Because of the intense black coloring of asphaltenes, it is impossible to estimate their solubility and distribution between paraffin and epoxy phases of emulsions using direct methods. Nevertheless, some conclusions about the miscibility and phase states of epoxy/paraffin/asphaltenes blends can result indirectly from data on their rheological and thermophysical behavior before, during, and after the curing.

### 3.2. Rheological Properties of Uncured Blends

**Figure 2:**
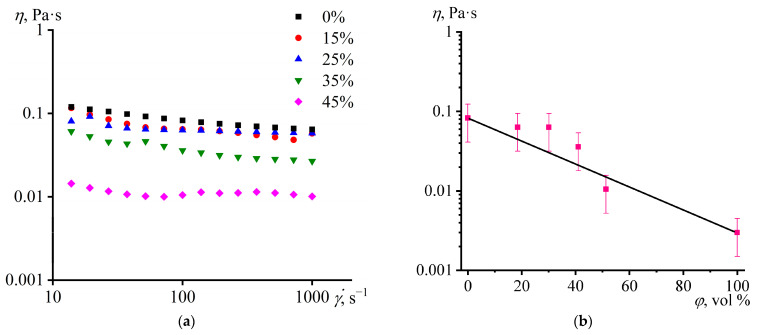
Dependences of viscosity on (**a**) shear rate and (**b**) paraffin wax concentration for epoxy compositions at 100 °C. The mass fraction of paraffin wax is indicated in the legend.

The mixing of epoxy resin, hardener, asphaltenes, and paraffin wax results in emulsions stable at high temperatures melting the wax. Note that asphaltene-free emulsions are unstable under the same conditions, indirectly confirming the role of asphaltenes as stabilizers. By measuring the flow curves of the uncured compositions under the mixing temperature of 100 °C, it turns out that their viscosity decreases as the concentration of paraffin increases (Figure 2a). The viscosity of mixtures usually obeys the rule of logarithmic additivity of components’ viscosities as follows [104,105]:

log*η*_12_ = *ϕ*_1_·log*η*_1_ + *ϕ*_2_·log*η*_2_, (7)

where *ϕ*_1_ and *ϕ*_2_ are the volume fractions of the components of the mixture with viscosities *η*_1_ and *η*_2_, respectively. In our case, this rule is satisfied in the first approximation, and the points of the experimentally measured viscosity approximately lie on the straight line connecting the logarithms of the viscosities of the pure molten wax and wax-free epoxy composition (Figure 2b). Usually, the negative deviation from the log-additivity rule is due to interfacial slip in immiscible blends, whereas the positive one occurs in miscible polymer blends due to macromolecular entanglements [105,106]. In this respect, the fulfillment of the rule occurs in immiscible blends with good interfacial adhesion, although it can also be in miscible blends without entanglements.

**Figure 3:**
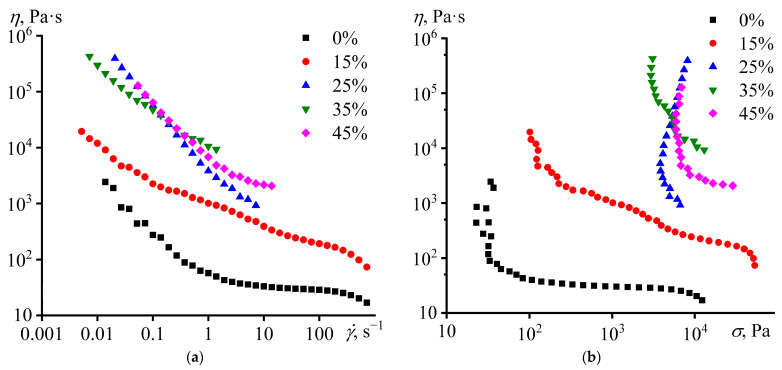
Dependences of viscosity on (**a**) shear rate and (**b**) shear stress for epoxy compositions at 25 °C. The mass fraction of paraffin wax is given in the legends.

Even wax-free base epoxy composition becomes a non-Newtonian fluid when cooled to 25 °C (Figure 3a). The shear-thinning behavior may result from the disagglomeration of the hardener particles, which initially were in an agglomerated state and formed a spatial network. An increase in the shear rate elevates the rate of the destruction of coagulation interparticle contacts versus the constant rate of their restoration, thereby reducing the viscosity of the dispersion [107]. In this case, the addition of paraffin wax increases the viscosity of the epoxy composition, which is expected because the wax droplets turn into solid particles at this temperature. However, the viscosity increases to a certain wax content of about 25%, and then the addition of wax has no significant effect. This assumes that crystalline wax particles can interact with hardener particles to embed into their structural network but only for this specific concentration without further influence on the network.

The presence of the structural network is confirmed when considering the dependences of viscosity on shear stress (Figure 3b); even the wax-free composition has a yield stress. It is expressed in the non-fluidity of the composition at shear stresses below 30 Pa, whereas when this stress is reached, the effective viscosity drops sharply, which looks like a vertical section of the curve. In this case, the yield stress value corresponds to the strength of the network from hardener particles. Paraffin wax increases the effective viscosity and yield stress of compositions markedly but up to certain wax content. The value of the yield stress of dispersions depends on the strength of interparticle contacts and their density. The density of interparticle contacts increases when the volume fraction of the particles increases or/and their size decreases. Since the yield stress practically does not change when the paraffin wax content grows in the range of 25–45%, it can conclude that either the size of the wax particles becomes higher, leading to an approximate invariance of the density of interparticle contacts despite the increase in the volume fraction of particles, or interparticle interactions become weaker, e.g., due to partial replacement of the initial hardener/hardener and hardener/wax particle interactions by wax/wax ones.

**Figure 4:**
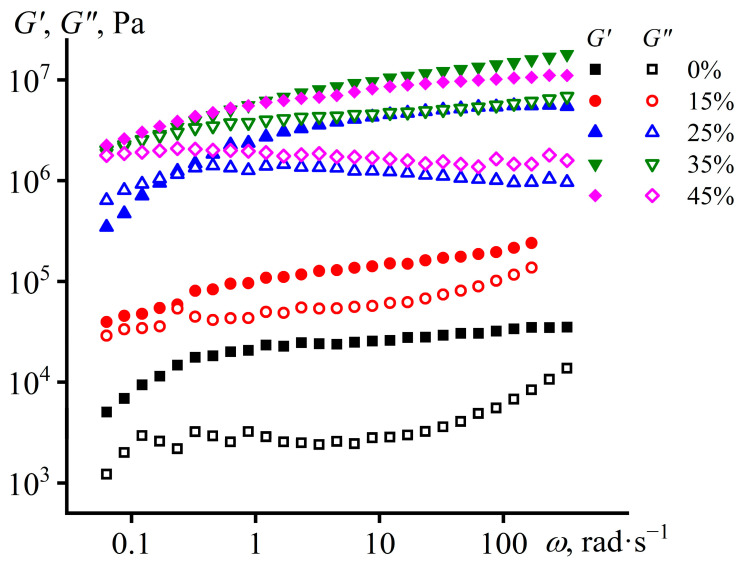
Frequency dependences of storage and loss moduli for epoxy compositions at 25 °C. The mass fraction of paraffin wax is given in the legend.

The study of viscoelastic properties can provide additional information about the structure of dispersed systems (Figure 4). The storage modulus of the wax-free epoxy composition exceeds its loss modulus and is weakly dependent on frequency, indicating a structured state of the system [108]. The addition of paraffin wax causes a progressive increase in both moduli. A larger quantity of solid particles provides a higher density of interparticle contacts and hence a greater stiffness of the resulting percolation structure. When the wax content reaches 45%, the growth of the storage modulus stops (the density of interparticle contacts remains unchanged), but the loss modulus decreases, i.e., there is a decrease in the tendency of the composition to irreversible deformations. For all systems, both moduli tend to decline with decreasing frequency, indicating the potential ability of the compositions to flow at very long observation times. In other words, these systems require chemical curing for practical use and form stability.

### 3.3. Curing of the Blend

**Figure 5:**
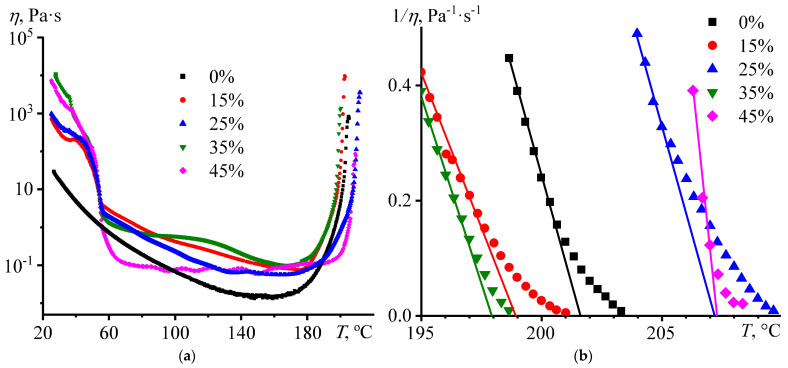
Variation of the effective viscosity in (**a**) normal and (**b**) reciprocal coordinates during the curing of epoxy compositions in the mode of gradual temperature increase at a rate of 2 °C/min and a shear rate of 1 s^−1^. The mass fraction of paraffin wax is given in the legends.

When curing the initial wax-free composition in the mode of gradual increase in temperature, the change in its viscosity goes through several stages (Figure 5a). In the first stage, the viscosity of the composition monotonically decreases in the range from 25 to 145 °C due to a decrease in the energy of intermolecular interactions. At 145–160 °C, the viscosity takes on an almost constant value, which can be explained by two processes that have an opposite effect on the viscosity value. On the one hand, an increase in temperature reduces viscosity due to a decrease in the energy of intermolecular interactions. On the other hand, the cross-linking of the epoxy resin with the hardener starts in this temperature range, accompanied by an increase in its molecular weight and hence the viscosity, and the cross-linking accelerates as the temperature becomes higher. The superposition of the two processes leads to a quasi-permanent viscosity, but it does change when the temperature rises due to a decrease in the energy of intermolecular interactions and an increase in the molecular weight of the epoxy medium. An increase in temperature above 160 °C causes the second stage of curing, accompanied by an intense increase in viscosity due to the acceleration of the curing and the dominance of this factor over the growth of thermal energy of the molecular movement. The viscosity increases to a temperature of 205 °C, after which the third stage of curing starts with a loss of fluidity of the composition, i.e., with the formation of a three-dimensional network of chemical bonds throughout the entire volume of the sample, meaning its gelation.

The paraffin wax affects the effective viscosity of compositions and its change during curing. In almost all cases, the wax increases the effective viscosity, especially at temperatures below 55 °C when it is in a solid state. The wax melting reduces the effective viscosity since the viscosity of an emulsion is lower than that of a suspension under comparable conditions [109], but it is still higher than the viscosity of the base wax-free system. The exception is the blend containing 45% wax, as its effective viscosity drops sharply when the wax melts and then remains virtually unchanged when the temperature rises until the gel point is reached. There is likely a wall slip of this blend rather than its flow between the measuring steel surfaces, perhaps due to the release of wax on the surface of the sample and the poor adhesion of paraffin wax and steel. At the same time, the effect of wax on the viscosity value at its content of 15–35% is not expressed clearly. It may result from the dependence of the effective viscosity on the solubility of molten wax in the continuous epoxy medium and on the size of its dispersed droplets, depending implicitly on the wax concentration and temperature.

The position of the gel point can be determined by extrapolating the inversed effective viscosity 1/*η* to zero (Figure 5b). In this case, the influence of paraffin wax is also not straightforward, and the position of the gel point can shift by 4–5 °C in both directions from the point inherent to the base composition (*T*_gel_, Table 2). On the one hand, the wax can accelerate the curing by dissolving in the epoxy medium, reducing its viscosity, and promoting the diffusion of the reacting substances. On the other hand, the dissolution of wax in the epoxy medium reduces the concentrations of the reacting substances and slows down cross-linking. These two effects should manifest themselves differently in the curing process. At the initial curing stage, the viscosity of the continuous medium is low, and the curing rate is determined primarily via the concentration of the reacting substances rather than their diffusion. In this case, the dissolution of paraffin wax slows down the cross-linking reaction, reducing the reagents’ concentrations. In the subsequent curing stages, the viscosity of the continuous phase grows sharply due to the increase in molecular weight of the epoxy polymer, and the overall curing rate is determined predominantly by the diffusion rate of the reagents. In this case, the dissolution of the wax reduces viscosity, speeding up the diffusion and the overall rate of cross-linking accordingly. This possible complex bidirectional effect of paraffin wax on the epoxy curing is likely the reason for the ambiguous displacement of the gel point. Indirect confirmation of this mechanism of paraffin action is a shift of the viscosity minimum towards higher temperatures when the wax is added (from 160 °C to 166–177 °C), i.e., the slowing down of the initial curing stage. In contrast, the gel point for half of the systems is shifted toward lower temperatures, indicating some acceleration of cross-linking in the second curing step.

**Figure 6:**
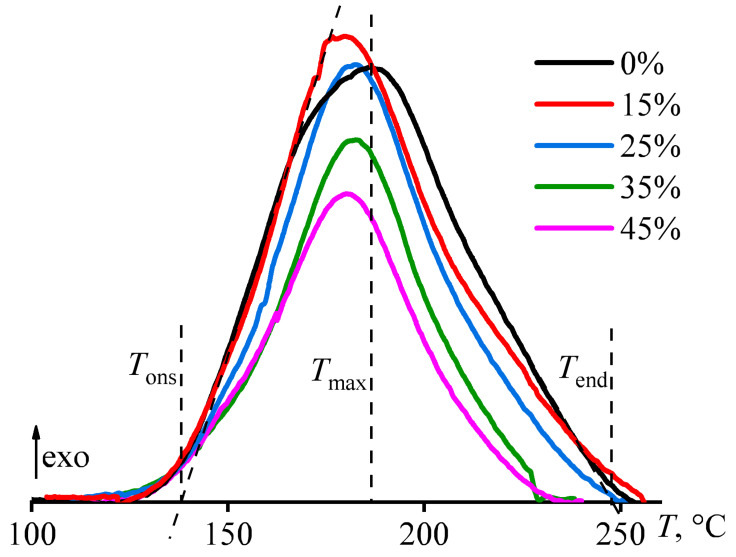
Change in heat flow during the curing of epoxy compositions at a heating rate of 2 °C/min. The mass fraction of paraffin wax is given in the legend.

The bidirectional effect of paraffin wax on the curing rate is also confirmed by data on the change in heat flux during cross-linking of the systems by a gradual increase in their temperature (Figure 6). The point of the nominal starting of cross-linking can be determined by the intersection of the baseline of the DSC curve and the tangent drawn to the line of monotonic growth in the heat release at the initial curing stage (*T*_ons_, Table 2). The paraffin wax slows down the initial curing rate, which is reflected in a shift of the heat release onset point toward higher temperatures by 3.2–7.0 °C. At the same time, the wax accelerates the curing at its second stage, judging by the position of the heat-release maximum temperature (*T*_max_), which shifts towards lower temperatures by 3.7–9.7 °C. The acceleration is also indicated by the nominal temperature of the end of cross-linking (*T*_end_), which can be defined as the point of intersection of the thermogram baseline and the tangent drawn to the heat flow curve at the final curing stage. The *T*_end_ position allows for estimating the total effect of wax addition on the overall curing rate of epoxy systems during all curing stages. A small wax content (*w*_wax_ = 15%) slightly slows cross-linking since *T*_end_ increases by 5 °C. On the contrary, higher concentrations of paraffin wax accelerate curing and cause a decrease in *T*_end_. Moreover, the higher the wax concentration, the lower *T*_end_, and the maximum decline is 21 °C.

As the mass fraction of paraffin wax increases, the overall heat effect of cross-linking declines due to a decrease in the proportion of epoxy resin and hardener in the blend (Δ*H*, Table 2). In this case, the completeness of the epoxy resin curing can be estimated using the reduced heat effect of cross-linking as follows:

Δ*H*_red_ = 100·Δ*H*/(100 − *w*_wax_ − *w*_asph_), (8)

where *w*_wax_ and *w*_asph_ are the mass fractions of paraffin wax and asphaltenes in the blend, respectively. The addition of 15–45% wax increases the reduced heat effect when compared to that of the base epoxy matrix, which can result from two factors. First, the wax may increase the curing depth due to partial solubility in the epoxy medium and improved diffusion in the final curing stage. Second, chemical reactions may be between the epoxy resin and asphaltenes containing reactive groups [66]. In turn, the change in the cross-linking degree should affect the thermophysical properties of these blends after their curing.

### 3.4. Thermophysical and Morphological Features of Cured Blends

**Figure 7:**
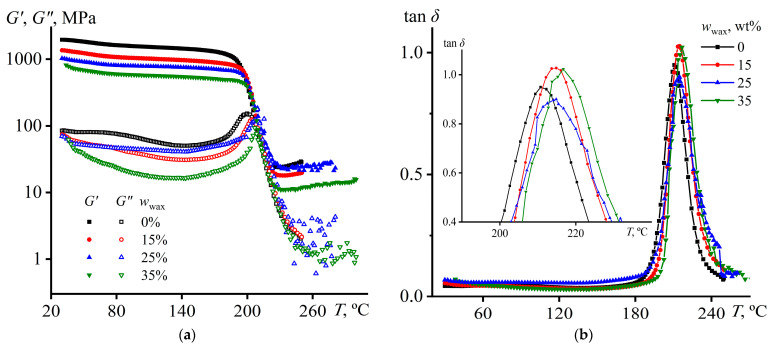
Temperature dependences of (**a**) the storage and loss moduli and (**b**) the loss tangent of epoxy polymer containing asphaltene-stabilized paraffin wax. The inset shows the enlarged area of the loss tangent maximum.

In general cases, a decrease in the completeness of epoxy curing is indicated in most by decreasing a glass transition temperature of a cured polymer. In our case, the study of cured samples using the DMA method shows the growth of their glass transition temperature with an increase in the content of asphaltene-stabilized wax (Figure 7). This conclusion results from the shifts of maxima of loss modulus and loss tangent towards higher temperatures and the similar change in the onset point of storage modulus drop. The drop in storage modulus occurs at lower temperatures (*T*_g,*G*′_, Table 3) than reaching the maximum of the loss modulus (*T*_g,*G*″_) or loss tangent (*T*_g,tan*δ*_). This dissimilarity in the *T*_g_ location is from the gradualness of glass transition due to the polydispersity of the initial epoxy molecules and the resulting unequal distance between the formed cross-links. Asphaltene-stabilized wax increases the glass transition temperature according to all three methods of its determination, which may indicate a higher cross-link density in the cured polymer. The second explanation may suggest that asphaltenes play the role of a filler, which adsorbs polymer chains, reducing their mobility and increasing the glass transition temperature [110,111,112,113].

Meanwhile, the storage modulus decreases with the addition of wax at both low (*G*′_30°C_) and high temperatures (*G*′_250°C_). In our case, the wax-containing cured polymers are heterogeneous systems having a continuous epoxy matrix and dispersed paraffin phase. Like the viscosity, the dynamic moduli of a heterogeneous mixture compose those of the dispersion medium and the dispersed phase according to some averaging law, most often the logarithmic additivity rule, i.e., the weighted geometric mean, as follows [96]:

log(*G*′_blend_) = *ω*_wax_ × log(*G*′_wax_) + (1 − *ω*_wax_) × log(*G*′_epoxy_). (9)

The flexural modulus of paraffin wax is about 150 MPa in the crystalline state [114], becoming a zero at 250 °C, which allows for calculating the theoretical stiffness of wax-containing mixtures according to Equation (9).

**Figure 8:**
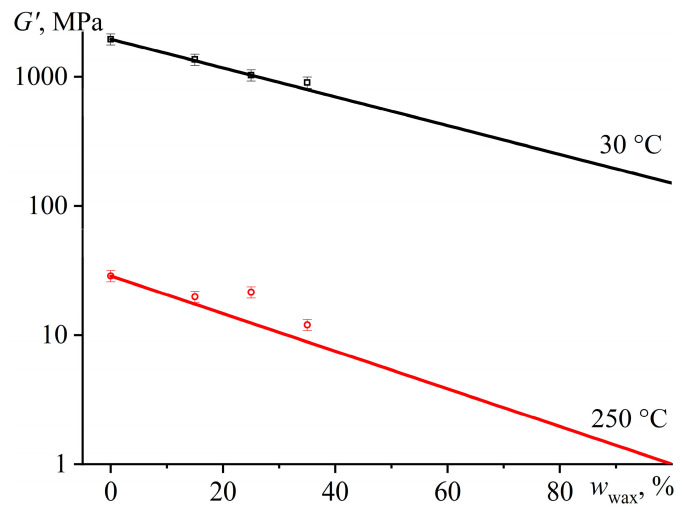
The concentration dependence of the storage modulus of epoxy polymer containing paraffin wax stabilized with asphaltenes. The dots show the experimental data, while the lines represent the log-additive rule.

A comparison of the experimentally measured storage modulus with the calculated data shows their good convergence at 30 °C when the cured specimens are in the glassy state (Figure 8). Thus, the log-additivity rule correctly describes the change in the viscoelasticity of epoxy polymer containing dispersed wax. However, the situation changes at 250 °C; the experimentally measured storage moduli are higher than the theoretical straight line. Note that according to viscosity tests, there were also positive deviations of the measured viscosity from the log-additivity rule (Figure 2b). However, the scatter of experimental viscosities was much larger, likely due to the heterogeneous state of the blends, and the deviation from a straight log-additivity line was within the measurement error (shown by the error bars in Figure 2b). When testing the cured specimens, the error was much smaller (the error bars in Figure 8), and therefore, the deviations of the storage modulus from the log-additivity line are significant. In addition, the deviations of the measured storage modulus are different at 30 °C and 250 °C for the same samples, which also indirectly confirms the significance of the positive deviations at 250 °C. Since the dispersed molten wax is inelastic, the additional elasticity can result only from a change in the epoxy matrix characteristics. The stiffness of a cured epoxy matrix at high temperatures exceeding the glass transition temperature (co-called the rubbery plateau modulus, $G_{N}^{0}$) connects directly with the cross-link density *q* as follows [115]:

(10) $$G_{N}^{0}=\frac{\rho k_{B}TN_{A}}{M_{e}}=\frac{\rho k_{B}TN_{A}M_{m}}{q},$$

where *ρ* is the material density, *N*_A_ is the Avogadro number, *k*_B_ is the Boltzmann constant, *T* is thermodynamic temperature, *M*_m_ is the monomer molecular weight, and *M*_e_ is the molecular weight between chemical cross-links (or macromolecular entanglements in the case of non-crosslinked polymer in the rubbery-plateau state). Thus, the increase in the rubbery plateau modulus is associated with a decrease in the molecular weight between the cross-links. There can be two explanations for this fact. First, there may be an increase in chemical cross-link density. Since paraffin wax cannot chemically interact with the epoxy resin, the rise in the cross-link density can only be from the asphaltenes, i.e., from their incorporation into the cross-linked gel network of the epoxy polymer. Second, asphaltene particles acting as filler may adsorb sections of polymer chains between the chemical cross-links, giving them the role of physical cross-links, increasing the overall cross-link density of the epoxy polymer and increasing its stiffness. Furthermore, asphaltene particles may increase the shear modulus in the glassy and rubbery states of the epoxy polymer because of their higher stiffness, like solid particles of usual fillers [116,117,118,119].

**Figure 9:**
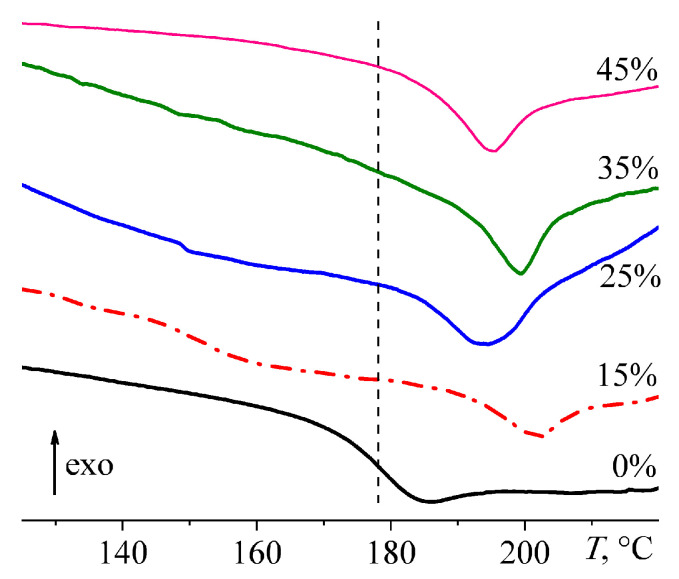
DSC thermograms of cured epoxy compositions in the glass transition region. The mass fraction of paraffin wax is indicated next to the curves. The vertical dashed line shows the position of the glass transition temperature for the base epoxy composition.

Additional indirect evidence for the increased cross-link density comes from calorimetry data. According to the thermogram of the cured wax-free epoxy polymer (Figure 9), its glass transition temperature is 178.2 °C (*T*_g_, Table 4). The addition of asphaltene-stabilized paraffin wax elevates the glass transition temperature to 190–197 °C, indicating more frequent chemical or/and physical cross-linking of compositions containing paraffin wax and asphaltenes. There may be two reasons for the denser network of chemical and physicochemical bonds, i.e., covalent and van der Waals ones, respectively, from epoxy/hardener reaction and epoxy/asphaltene adsorption. First, the wax could contribute to a completer chemical reaction between the epoxy resin and the hardener due to the plasticizing effect and improved diffusion of the reacting components in the later curing stages. However, this conclusion is questionable because plasticization should decrease the glass transition temperature, which is not observed experimentally. Second, the density of the cross-links network could have increased due to the integration of asphaltenes that can form covalent and non-covalent bonds with the epoxy matrix [66], which seems more likely. In any case, the glass transition temperature exceeds the planned operating temperatures of the phase-change material (about 30–60 °C) and thus provides it with form stability.

**Figure 10:**
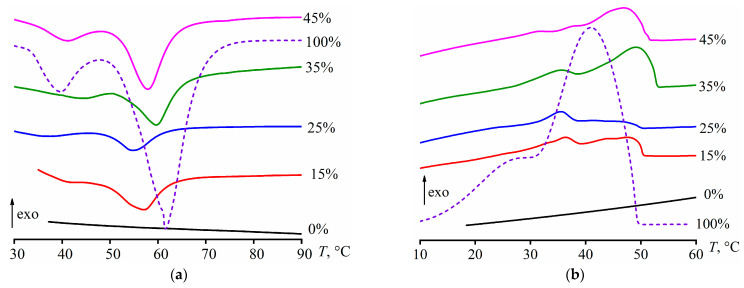
DSC thermograms at (**a**) heating or (**b**) cooling of cured epoxy compositions in the regions of melting and crystallization of paraffin wax, with mass fraction indicated next to the curves.

The operating temperature range of the PCM should be close to the temperatures of crystallization and melting of paraffin wax, which determines the heat-accumulating properties of the compositions. Two transitions of pure paraffin wax can be detected on its thermograms (Figure 10). The weak low-temperature transition at 30–40 °C is due to the polymorphic transformation of the wax from one crystalline state to another [120]. This transformation also can serve a practical function of thermal energy storage. The larger-energy solid–liquid transition occurs at higher temperatures, resulting in the following: melting at 61.7 °C (*T*_m_, Table 4) and crystallization at 40.8 °C (*T*_cr_). The dispersing of paraffin wax in the asphaltene-containing epoxy matrix reduces its melting point to 54–60 °C. It can be explained by a more defective structure of wax crystals due to asphaltene impurities, which entered the wax phase because of the limited miscibility (see Figure 1). In addition, the asphaltene impurities act as nucleators for paraffin molecules since they crystallize at higher temperatures (46–49 °C).

Based on the enthalpies of melting (Δ*H*_m_) and crystallization (Δ*H*_cr_) of cured compositions, it is possible to calculate a nominal degree of crystallinity of dispersed paraffin wax as follows:

(11) $$\mathrm{DC}=\frac{100\left(\Delta H_{\mathrm{cr}}+\Delta H_{m}\right)}{w_{\mathrm{wax}}\left(\Delta H_{\mathrm{cr},\mathrm{wax}}+\Delta H_{m,\mathrm{wax}}\right)}\cdot 100\%,$$

where *w*_wax_ is the mass fraction of paraffin wax in the cured composition, while Δ*H*_m,wax_ and Δ*H*_cr,wax_ are the enthalpies of melting and crystallization of pure wax, respectively. Calculations show that the crystallinity degree of paraffin wax decreases sharply to 13–29% of the initial value when it is in the epoxy matrix (DC, Table 4). There are four possible reasons for dropping the wax crystallinity. First, some of the wax may dissolve in the epoxy matrix. Second, there can be a partial dissolution of asphaltenes in the dispersed paraffin wax and suppression of its crystallization. Third, partial dissolution of epoxy resin in the dispersed wax is also possible with a reduction in the wax’s crystallinity. Fourth, a decrease in crystallinity may result from the small size of wax particles formed from molten wax droplets.

**Figure 11:**
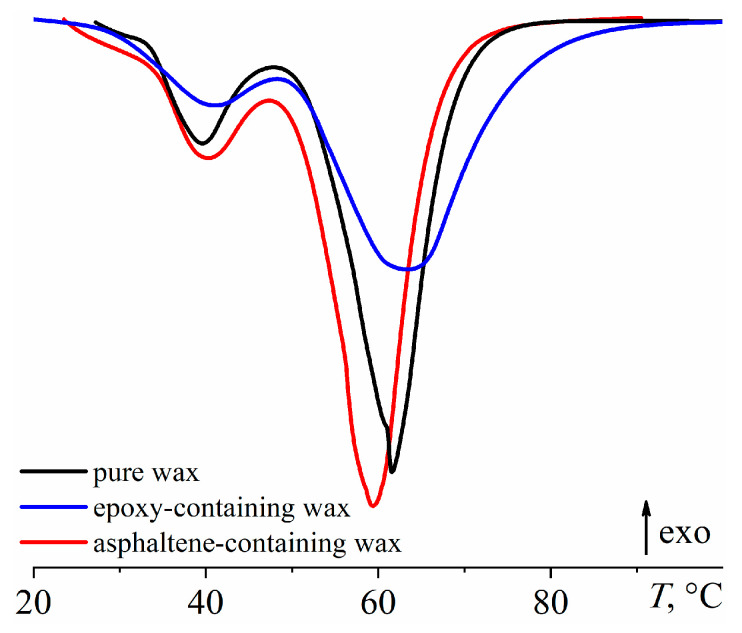
DSC thermograms when heating pure paraffin wax or paraffin wax containing asphaltenes or epoxy resin (their saturated solutions).

First of all, it is difficult to expect that paraffin wax noticeably dissolved in the cured epoxy medium since then it would act as a plasticizer and lower the glass transition temperature of the epoxy polymer, which is not observed experimentally (see Table 4, *T*_g_). For testing the second hypothesis of suppression of wax crystallinity with asphaltenes, their saturated solution was prepared by adding 10% asphaltenes to the pure paraffin wax and stirring them at 180 °C. A comparison of the thermograms of the obtained asphaltene-containing wax and the initial pure wax shows that asphaltenes do not suppress crystallization in any noticeable way (Figure 11, peak area remains unchanged) but slightly reduce the wax melting temperature to 59.4 °C. It should be noted that the wax has a lower melting point in the epoxy matrix (57.3–59.6 °C, Table 4), which may be due to its dispersed state or the influence of impurities of epoxy resin rather than asphaltenes.

For testing the third hypothesis of the resin impurities’ influence, the saturated solution of the epoxy resin in paraffin wax was prepared. The resin and wax were mixed at a ratio of 1/1 at 180 °C, then the resulting blend was allowed to stand at this temperature, separating into two layers, cooled down to 25 °C, and the upper wax-rich layer was taken. According to the thermograms (Figure 11), the resin impurities cause a slight increase in the wax’s melting temperature to 63.4 °C. However, the total area of the endothermic peaks remains unchanged within the measurement error despite their substantial visual transformation. Thus, there is no change in the crystallinity degree. In this case, the melting peak widens toward higher temperatures, i.e., the epoxy resin contributes to less defective and higher-melting paraffin crystals.

**Figure 12:**
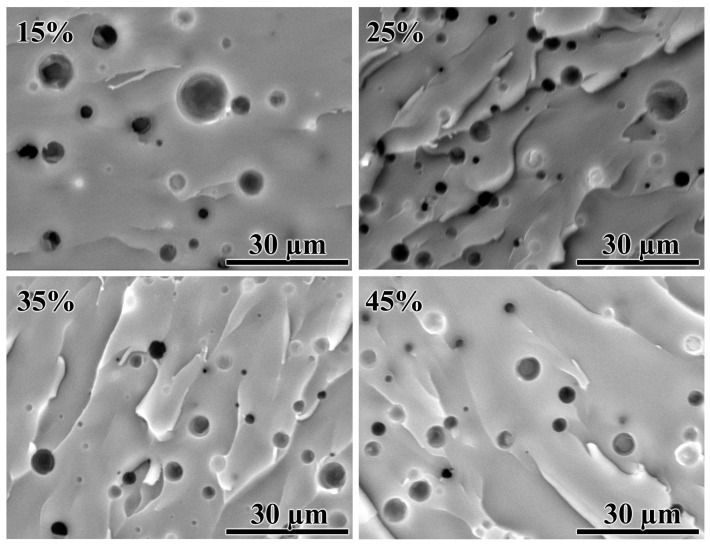
SEM images of cross-sections of cured epoxy compositions containing paraffin wax, with mass fraction indicated in the top-left corners.

**Figure 13:**
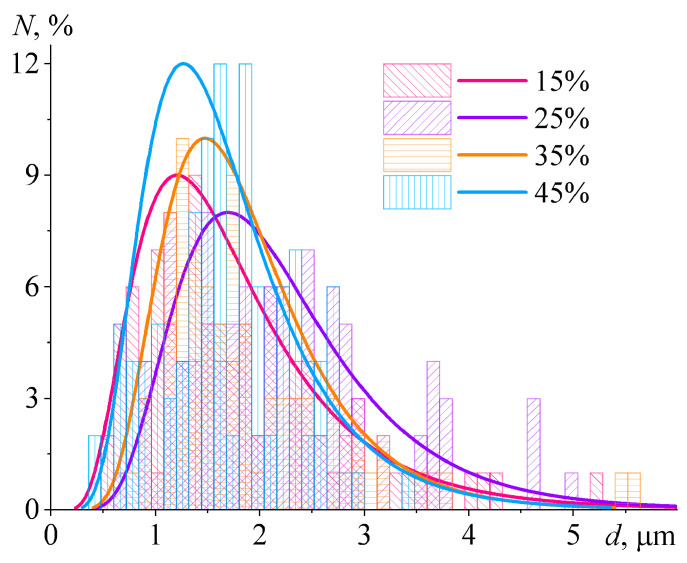
Number-average size distribution of paraffin wax droplets according to the analysis of SEM data. The histograms represent the measured diameters, while the curves approximate them according to a log-normal distribution. The paraffin wax mass fraction is given in the legend.

The fourth version requires an assessment of the morphology of cured compositions (Figure 12). It turns out that paraffin wax forms droplets with sizes from 0.2 μm to 6.5 μm in the epoxy matrix. In this case, the visible number of droplets and their average size do not depend appreciably on the wax content (Figure 13 and Table 5). A droplet size of 1–2 µm is large enough to influence the crystallinity degree of wax or its melting point. However, tiny wax droplets of 200–300 nm in diameter also occur. At the same time, if the size of a polymer phase is reduced to the nano- or submicron scale, its crystallization temperature is highly decreased by up to 40 °C in down [121]. It can be assumed that this effect is similarly inherent to paraffin wax, given its oligomeric nature and the large size of molecules. Tiny droplets of molten wax are closed systems where large crystals cannot grow up due to the limited volume. Under free space conditions, the paraffin wax forms needle-like crystals with a length of more than 100 µm [13], which obviously cannot grow inside droplets of submicron diameter. This means that the decrease in the crystallinity degree of paraffin wax is most likely due to the small size of its droplets in the epoxy matrix.

## 4. Conclusions

The study of rheological and thermophysical properties of phase-change materials based on epoxy compositions containing different amounts of asphaltene-stabilized paraffin wax before, during, and after their curing revealed the following:

- Epoxy resin and paraffin wax are poorly miscible even during high-temperature curing at 180 °C, which leads to a two-phase curing mixture (a wax-in-resin emulsion). In turn, asphaltenes dissolve in both phases at high temperatures, entering them and stabilizing the molten wax droplets.
- Uncured blends are non-Newtonian fluids with yield stress at 25 °C due to a structural network from hardener and paraffin wax particles. The network disappears at heating due to wax melting, causing the blends to become Newtonian fluids.
- The paraffin wax increases the viscosity of the epoxy resin up to 10 times but has no pronounced effect on the cross-linking rate and its completeness. At the same time, the asphaltenes may be involved in a chemical reaction with the epoxy resin, increasing the thermal effect of curing.
- Although the paraffin wax does not plasticize the cured epoxy matrix, it does not fully crystallize due to its small dispersed size.

An advantage of the obtained systems is the stabilization of paraffin/epoxy emulsions using asphaltenes as only surfactants. It opens up a new opportunity for the rational disposal of asphaltenes left from the deasphalting of heavy crude oil as part of new energy-saving materials. However, the disadvantage of these materials is the reduced crystallinity of the dispersed paraffin wax, which lowers the efficiency of their heat storage capacity. In turn, this suggests a direction for further work on other phase-change agents with a higher ability to crystallize as a dispersed phase of epoxy phase-change material, perhaps due to the lower molecular weight or larger particle size. Further work will also require a more rigorous assessment of the variability in the efficiency of the phase-change material due to multiple heating/cooling cycles and studies of their thermal conductivity.

## Figures and Tables

**Table 1:** Formulations of base epoxy matrix and epoxy phase-change materials.

#	Epoxy resin, wt%	Hardener, wt%	Paraffin wax, wt%	Asphaltenes, wt%
1	74.4	25.6	0	0
2	62.1	21.4	15	1.5
3	53.9	18.6	25	2.5
4	45.8	15.7	35	3.5
5	37.6	12.9	45	4.5

**Table 2:** Temperatures of curing onset, heat-release maximum, gel formation, and curing end for epoxy compositions containing different mass fractions of paraffin wax, as well as the measured and reduced heat effects of their curing.

*w*_wax_, wt%	*T*_ons_, °C	*T*_max_, °C	*T*_gel_, °C	*T*_end_, °C	Δ*H*, J/g	Δ*H*_red_, J/g
0	140.3	186.3	202	248	300	300
15	143.5	176.6	199	253	294	352
25	147.3	182.6	207	243	267	368
35	146.6	182.6	198	231	195	318
45	143.8	180.1	207	227	173	342
Standard deviation	0.3	0.2	1	1	15	15

**Table 3:** The glass transition temperature and storage modulus of epoxy polymer containing asphaltene-stabilized paraffin wax.

*w*_wax_, wt%	*T*_g,*G*′_, °C	*T*_g,*G*″_, °C	*T*_g,tan*δ*_, °C	*G*′_30°C_, MPa	*G*′_250°C_, MPa
0	184.9	198.7	210.7	1960	28.7
15	192.4	204.0	214.9	1360	19.8
25	194.4	207.1	214.8	1030	21.5
35	196.7	206.8	216.7	904	12.0
Standard deviation	0.2	0.2	0.2	10%	10%

**Table 4:** The glass transition temperatures of cured compositions containing paraffin wax with indicated temperatures and enthalpies of crystallization and melting and degree of crystallinity.

*w*_wax_, wt%	*T*_g_, °C	*T*_cr_, °C	*T*_m_, °C	Δ*H*_cr_*,* J/g	Δ*H*_m_, J/g	DC, %
0	178.2	-	-	-	-	-
15	196.6	47.6	57.3	7.8	8.4	28.7
25	189.8	46.1	54.0	4.4	7.4	12.5
35	193.9	49.1	59.6	13.6	13.1	20.2
45	191.7	47.4	58.0	12.0	20.0	18.9
100	-	40.8	61.7	195	182	100
Standard deviation	0.2	0.2	0.2	5%	5%	5

**Table 5:** The average diameter of paraffin wax droplets in the cured epoxy compositions.

*w*_wax_, wt%	Droplet Diameter, μm
15	1.8 ± 0.9
25	2.2 ± 0.9
35	1.9 ± 0.9
45	1.7 ± 0.6

## Data Availability

The data presented in this study are available upon request from the corresponding author.
